# Biomechanical analysis and clinical observation of 3D-printed acetabular prosthesis for the acetabular reconstruction of total hip arthroplasty in Crowe III hip dysplasia

**DOI:** 10.3389/fbioe.2023.1219745

**Published:** 2023-09-15

**Authors:** Yuchen Liu, Fuyang Wang, Jiawei Ying, Minghao Xu, Yuan Wei, Junlei Li, Hui Xie, Dewei Zhao, Liangliang Cheng

**Affiliations:** ^1^ Department of Orthopedics, Affiliated Zhongshan Hospital of Dalian University, Dalian, China; ^2^ Affiliated Zhongshan Hospital of Dalian University, Dalian, China

**Keywords:** developmental dysplasia of the hip, acetabular reconstruction, 3D printing, integral acetabular prosthesis, modular acetabular prosthesis, finite element analysis

## Abstract

**Objective:** This study aimed to evaluate the biomechanical effectiveness of 3D-printed integrated acetabular prosthesis (IAP) and modular acetabular prosthesis (MAP) in reconstructing the acetabulum for patients with Crowe III developmental dysplasia of the hip (DDH). The results of this study can provide a theoretical foundation for the treatment of Crowe III DDH in total hip arthroplasty (THA).

**Methods:** Finite element (FE) analysis models were created to reconstruct Crowe III DDH acetabular defects using IAP and MAP. The contact stress and relative micromotion between the acetabular prosthesis and the host bone were analyzed by gradually loading in three increments (210 N, 2100 N, and 4200 N). In addition, five patients with Crowe III DDH who underwent IAP acetabular reconstruction were observed.

**Results:** At the same load, the peak values of IAP contact stress and relative micromotion were lower than those of MAP acetabular reconstruction. Under jogging load, the MAP metal augment’s peak stress exceeded porous tantalum yield strength, and the risk of prosthesis fracture was higher. The peak stress in the bone interface in contact with the MAP during walking and jogging was higher than that in the cancellous bone, while that of IAP was higher than that of the cancellous bone only under jogging load, so the risk of MAP cancellous bone failure was greater. Under jogging load, the relative micromotion of the MAP reconstruction acetabular implant was 45.2 μm, which was not conducive to bone growth, while under three different loads, the relative micromotion of the IAP acetabular implant was 1.5–11.2 μm, all <40 μm, which was beneficial to bone growth. Five patients with IAP acetabular reconstruction were followed up for 11.8 ± 3.4 months, and the Harris score of the last follow-up was 85.4 ± 5.5. The imaging results showed good stability of all prostheses with no adverse conditions observed.

**Conclusion:** Compared with acetabular reconstruction with MAP, IAP has a lower risk of loosening and fracture, as well as a better long-term stability. The application of IAP is an ideal acetabular reconstruction method for Crowe III DDH.

## 1 Introduction

Development dysplasia of the hip (DDH) is characterized by abnormalities in the anatomy of the acetabulum and femur. This anatomical abnormality increases the contact stress of the hip joint, resulting in hip instability, impingement, and pathological state of the labrum, and finally develops into osteoarthritis (Gala et al., 2016). Osteoarthritis, secondary to hip dysplasia, includes thinning of articular cartilage, narrowing of joint space, subchondral cystic lesions, and even hip joint deformation. The clinical manifestations are hip joint pain and limited activity, which seriously affect the quality of daily life (Garcia et al., 2022). Although there are several alternatives to hip preservation, many adult patients with DDH eventually require hip replacement (Schmitz et al., 2020).

Total hip arthroplasty (THA) can relieve hip pain symptoms and improve hip function in patients with DDH and is the main surgical procedure for adult DDH (Zhang et al., 2022). Based on the subluxation height relative to the inter-teardrop line, DDH was divided into four types according to Crowe’s classification. The acetabular morphological deformity in Crowe III is more obvious than those in Crowe types I, II, and IV, resulting in more incredible difficulty of acetabular reconstruction and the installation of the acetabular prosthesis in THA, especially for the restoration of the hip rotational center and the reconstruction of bone defect (Wen et al., 2021). The traditional methods mainly include bone grafting, high hip center, and medial protrusio technique (Mou et al., 2020). However, the structural bone graft has problems, such as bone resorption and collapse (Goto et al., 2021). The internal or upward movement of the rotation center has the disadvantages of offset reduction and leg length discrepancy (Kleemann et al., 2003; Liu et al., 2018; Mou et al., 2020). For Crowe III DDH, choosing a more appropriate acetabular reconstruction method is an urgent issue for clinicians.

In recent years, with the development of the 3D printing technology, personalized orthopedic implant devices have provided solutions to many problems (Mirkhalaf et al., 2023). The 3D printing technology originated in the 1980s can provide preoperative visual and tactile evaluation and prepare individualized prostheses for different degrees and parts of orthopedic injuries, thus achieving improved surgical outcomes and reduced postoperative complications (Lee et al., 2020; Pu et al., 2021). Meanwhile, the 3D printing technology provides a new idea for acetabular reconstruction in patients with Crowe III DDH, which can personalize the design of metal augments and acetabular cups according to the acetabular bone defects in Crowe III DDH patients. We use screws to fix the metal augment on the bone defect and place the acetabular cup, which not only ensures the complete coverage of the acetabular cup but also restores the hip center of rotation and achieves the biomechanical stability of the acetabular cup (Zhang et al., 2020). However, the modular acetabular prosthesis (MAP) with multiple components (acetabular cup + metal augment + metal screw) poses a risk of inter-component failure (Strahl et al., 2023). To reduce the complex intra-operative manipulation and the potential of prosthetic loosening for the MAP, the integrated acetabular prosthesis (IAP) designed by the 3D printing technology may achieve better initial and long-term stability. This could effectively reduce the incidence of adverse events after acetabular reconstruction in patients with Crowe III DDH.

Additionally, selecting the appropriate acetabular prosthesis material is a key factor to the success of the surgery. Porous tantalum is currently the ideal orthopedic implant material for prosthesis repair. Its low modulus of elasticity prevents stress-shielding; a high coefficient of friction enhances the initial stability of the prosthesis; and the design of a bone trabecular structure promotes the ingrowth of new bone tissue (Junlei Li et al., 2020). Therefore, porous tantalum, which has more stable physicochemical properties, superior biomechanical performance, and better osseointegration ability, was chosen as the material for the acetabular prosthesis in this study.

Furthermore, the differences between the two acetabular prostheses regarding adaptability and biomechanical properties will be verified. In this study, a model of the acetabular bone defect of Crowe III DDH was established to simulate THA, and the initial stability of IAP and MAP acetabular reconstruction under different loads was compared and analyzed, which provides a reference for clinical selection of appropriate acetabular reconstruction from a biomechanical perspective.

## 2 Materials and methods

### 2.1 Establishment of the acetabular bone defect model of Crowe III DDH

**FIGURE 1:**
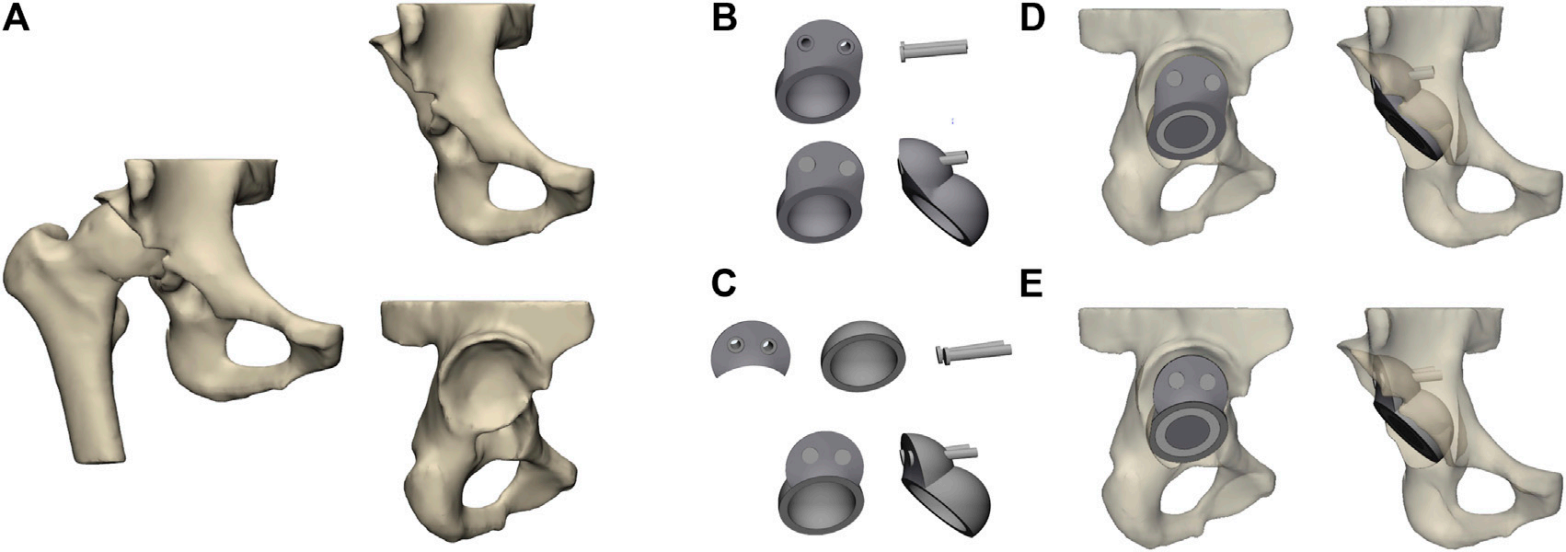
Establishment and assembly of models. **(A)** Geometrical model of the hip joint of the Crowe III developmental dysplasia of the hip (DDH); **(B)** integrated acetabular prosthesis (IAP) components; **(C)** modular acetabular prosthesis (MAP) components; **(D)** assembly of IAP acetabular reconstruction; **(E)** assembly of MAP acetabular reconstruction.

The subject, a 60-year-old man (175 cm; 70 kg) with Crowe III DDH, agreed and signed the informed consent form. A Siemens 64-row spiral CT scanner was used to scan the hip with a thickness of 0.5 mm. The CT image was stored in the standard Digital Imaging and Communications in Medicine (DICOM) format in Mimics 21 (The Materialise Group, Leuven, Belgium), a medical 3D reconstruction software. Appropriate gray values were selected to distinguish bone and tissue, and the three-dimensional model of the original hip was established (Wang et al., 2021) (Figure 1A). Then, the reconstructed model was imported into 3-Matic (The Materialise Group, Leuven, Belgium) software for surface optimization processing, such as model surface defect repair, smoothing, and accurate surface function.

### 2.2 Establishment and assembly of the acetabular prosthesis model

According to the size of the acetabulum of the subject, the acetabular cup component of the MAP was constructed in CAD (SolidWorks 2016; SolidWorks Corp, United States), and the diameter of the acetabular cup designed in this study was 50 mm. The anteversion angle of the acetabular cup was adjusted to 15° and the abduction angle was 45° (Pour et al., 2023). In CAD software, the IAP and metal augment were designed according to the acetabular bone defect’s size after the acetabular cup’s placement. Two screws (length: 35 mm and diameter: 6.0 mm) were used to fix the IAP and metal augment (Figures 1B, C). The parameters of the femoral head prosthesis were designed according to the diameter of the femoral head, and the 32-mm femoral ceramic head and corresponding polyethylene liner were implanted. Since the femoral part was not involved in this study, to save the calculation time of the Finite element (FE) model, the construction of the femoral and femoral stem prosthesis was omitted in this paper. In addition, to facilitate the application of the load, the femoral head of the prosthesis was simplified to a hemisphere in FE analysis (Wang et al., 2022). Finally, the aforementioned model was non-fluid-assembled in 3-Matic (Figures 1D, E).

### 2.3 Establishment of the FE model

**FIGURE 2:**
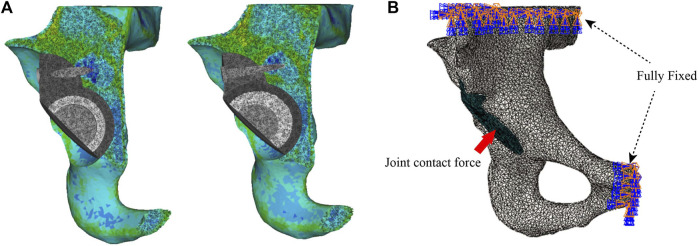
Setting of material properties, boundary conditions, and loads. **(A)** Setting the material properties of the iliac and acetabular prostheses and **(B)** loading and boundary conditions of FE modeling.

FE model was meshed with tetrahedral 4-node elements (C3D4). To obtain the actual structure and calculation proportion of the model, the mesh size was set to 1 mm, which has been validated by Dutt (2015). All models being analyzed were assumed to be continuous, isotropic, and with homogeneous linear elastic materials. The model was re-imported into Mimics 21, and material assignments were assigned according to the corresponding areas of the cortical and cancellous bone obtained by CT scanning (Guo et al., 2022) (Figure 2A). Table 1 lists the parameters of various materials (Fu et al., 2018).

**TABLE 1:** Material properties defined in the finite element (FE) models.

Components	Materials	Elastic Modulus (MPa)	Poisson’s ratio (*v*)
Cortical bone	Cortical bone	17,300	0.265
Cancellous bone	Cancellous bone	400	0.2
Screws	Titanium alloy	110,600	0.326
Acetabular cup	Tantalum	8,963	0.31
Metal augment			
Integrated cup			
Ceramic femoral head	Ceramics	350,000	0.22
Liner	Polyethylene	800	0.45

### 2.4 Setting of the model parameters

The previously assembled models were imported into Abaqus 2021 (Simulia Corp, Providence, RI, United States). Based on the previous studies setting frictional contact interactions, the friction coefficient between the bone–metal augment interface and the bone–acetabular cup interface was set to 0.8, the interface between the acetabular cup and the metal augment was established to a non-frictional connection, and the rest of the interfaces were tied connection (Du et al., 2020). In addition, fixed constraint boundary conditions were set on the pubis and the superior part of the ilium to prevent the model from moving during the analysis (Akrami et al., 2018) (Figure 2B). Based on the results of peak stresses in the unilateral hip joint reported in most of the literature, the hip contact force is 30% BW for double-legged standing (Xiong et al., 2022), 300% BW for walking, and 600% BW for jogging (Bergmann et al., 2001; Kitamura et al., 2022). The body weight of the volunteers was 70 kg. Therefore, in this study, we applied loads of 210 N, 2100 N, and 4200 N of hip contact forces to the rotation center of the femoral head (Soloviev et al., 2023).

### 2.5 Clinical application of the IAP in the acetabular reconstruction of Crowe III DDH

General data of patients: After obtaining the approval of the institutional ethics committee of the Affiliated Zhongshan Hospital of Dalian University, we performed a retrospective study that enrolled five patients with Crowe III DDH treated with 3D-printed porous tantalum IAP for THA in our hip joint department from January 2021 to January 2022. There were one male and four female patients with an average age of 65.2 ± 8.5 years.

Inclusion criteria: 1) Diagnosis of Crowe III DDH complicated with hip osteoarthritis and 2) patients agreed to hip replacement and signed the informed consent form.

Exclusion criteria: 1) The ages of the patients were below 30 years or above 80 years and 2) the primary diseases of the hip joint were other autoimmune diseases, infectious arthritis, or neoplastic conditions.

**FIGURE 3:**
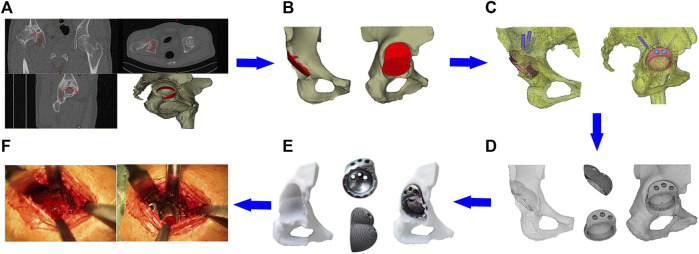
Clinical application of the IAP. **(A)** Acetabular cup placement in true acetabular position; **(B)** establishment of the integrated acetabular cup model; **(C)** design of the nail hole and screw direction; **(D)** porous treatment of IAP model; **(E)** preoperative model adaptation analysis; **(F)** surgical procedure.

Preoperative design and preparation of the prosthesis: The CT data of the patient’s hip joint were reconstructed on a computer, and the position of the acetabular cup was simulated in the true acetabulum position. The superolateral bone defect of the acetabular cup was filled with a sphere, forming an interconnected double spherical structure matched with the true and false acetabulum. A smooth curved transition between the edge of the acetabular prosthesis and the outer plate of the iliac crest should be carried out to avoid excessive protrusion of the outside of the augmentation device and preserve the nail hole position. The screw diameter, length, and fixation direction were designed according to the simulation design, defect location, and amount of residual bone. To avoid stress-shielding, the final structure of the IAP model was made porous by Magics (Materialise, Belgium). Finally, we imported the porous IAP model data into the 3D printer and used the laser powder bed fusion technology to prepare the prosthesis using tantalum powder (Figures 3A–E).

Surgical procedure: After the patient was anesthetized, the operation was performed via the posterolateral approach with the patient in the lateral decubitus position. The exposure and preparation of the acetabulum were the same as that for the posterolateral THA. The fibrotic scar tissue, thickened joint capsule, and surrounding hyperplastic osteophyte were removed intraoperatively. The acetabular reamer file was used to grind it to the preoperative planned size in the true and false acetabulum position. The 3D printing IAP was implanted and fixed with metal screws, finally installing the inner lining and femoral head prosthesis. The range of motion was checked adequately after hip reduction and before closing the incision (Figure 3F).

Postoperative management: The antero-posterior projection X-ray of the hip after the operation showed that the acetabular prosthesis was well-positioned with appropriate abduction and anteversion angle. Antibiotics were dripped for infection prophylaxis within 24 h. After resuscitation from anesthesia, patients should have ankle flexion and extension activities and quadriceps isometric muscle strength training under guidance. Partial weight-bearing exercise was allowed 24–48 h after the surgery, and the full weight-bearing exercise was permitted 2 weeks post-surgery.

### 2.6 Evaluation criteria

First, the stability of two acetabular prostheses in the Crowe III DDH acetabular reconstruction was evaluated by the contact stress and relative micromotion between the acetabular prosthesis and the host bone. Second, for the selected cases of the acetabular IAP reconstruction for Crowe III DDH in this article, we used the Harris hip score to evaluate hip joint function and used imaging examination to assess whether there were transparent line, displacement, aseptic loosening, osteolysis, and bone growth between the prosthesis and the bone surface.

## 3 Results

### 3.1 Validation of the developed FE model

The FE model used in this study for MAP acetabular reconstruction under walking and jogging loads had been validated through comparison with previous biomechanical models. The results of implant peak stress from Fu et al. (2018) (50.25 MPa for walking and 75.86 MPa for jogging) and relative micromotion under walking load (12.61 μm) from Wang et al. (2022) were compared with those of our study: implant peak stress of 52.3 MPa (walking) and 83.1 MPa (jogging), along with a relative micromotion of 13.7 μm under walking load. The results are similar, verifying that our FE model is suitable for further analysis.

### 3.2 Comparison of stress distribution in contact between the IAP and MAP

**FIGURE 4:**
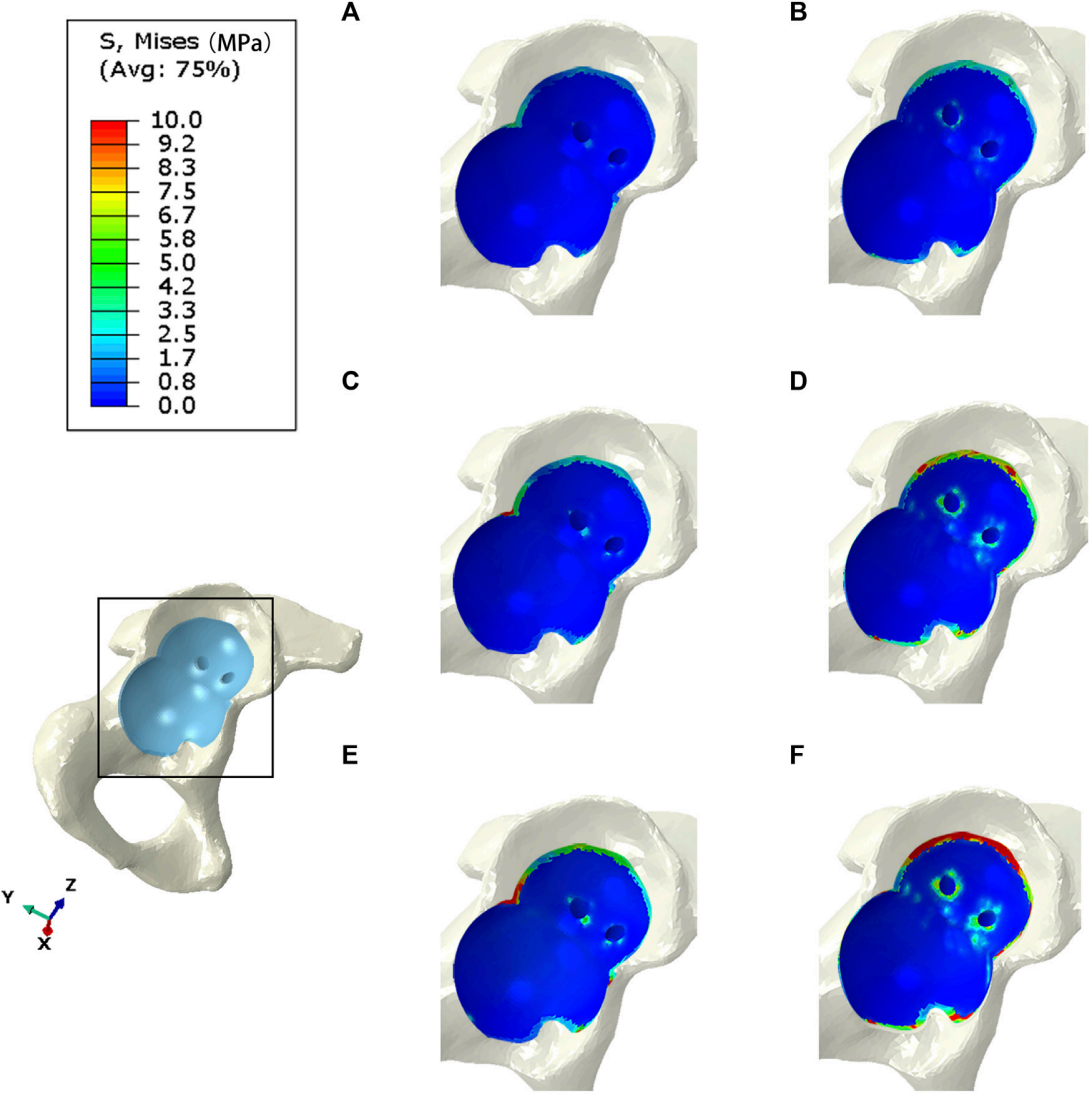
Stress distribution of bones contacted by IAP and MAP. **(A,B)** 210 N; **(C,D)** 2100 N; **(E,F)** 4200 N.

The bone interface in contact with the acetabular prosthesis is divided into the cortical bone and cancellous bone. The peak stress of the cortical bone interface was located at the edge of the cortical bone in contact with the acetabular prosthesis, and the peak stress of the cancellous bone interface was situated at the junction of the cancellous bone and the end edge of the metal screw. The peak stress at the cortical bone interface in contact with the IAP was 5.5 Mpa (210 N), 10.7 Mpa (2100 N), and 22.4 Mpa (4200 N), and the peak stress at the cancellous bone interface was 1.6 Mpa (210 N), 2.7 Mpa (2100 N), and 5.6 Mpa (4200 N) (Figures 4A, C, E). The peak stress at the cortical bone interface in contact with the MAP was 5.7 Mpa (210 N), 12.6 Mpa (2100 N), and 25.8 Mpa (4200 N), and the peak stress in the cancellous bone interface was 2.2 Mpa (210 N), 3.5 Mpa (2100 N), and 6.8 Mpa (4200 N) (Figures 4B, D, F).

### 3.3 Comparison of stress distribution between IAP and MAP implants

**FIGURE 5:**
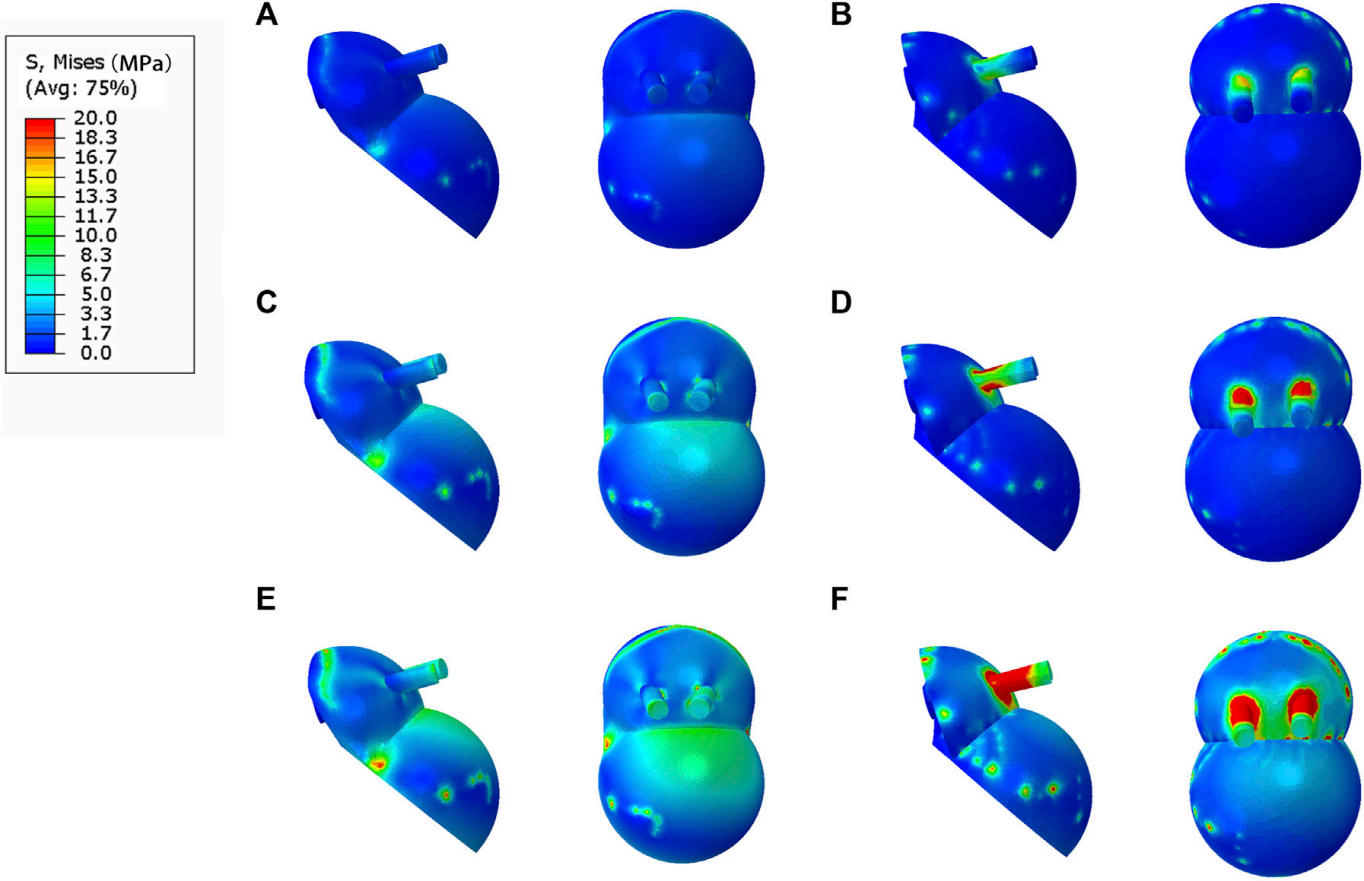
Stress distribution of IAP and MAP implants. **(A,B)** 210 N; **(C,D)** 2100 N; **(E,F)** 4200 N.

The peak stress of the IAP implant was located in the part of contact with the acetabular cortical bone, which was 12.7 Mpa (210 N), 16.2 Mpa (2100 N), and 25.8 Mpa (4200 N) (Figures 5A, C, E). The peak stress of the MAP implant was located at the lower one-third portion of the screw of the fixed metal augment, which was 17.6 Mpa (210 N), 52.3 Mpa (2100 N), and 83.1 Mpa (4200 N). The peak stress of the MAP metal augment was located at the nail hole, which was 12.2 Mpa (210 N), 20.6 Mpa (2100 N), and 54.2 Mpa (4200 N) (Figures 5 B, D, F).

### 3.4 Comparison of the relative micromotion of the IAP and MAP concerning the host bone

**FIGURE 6:**
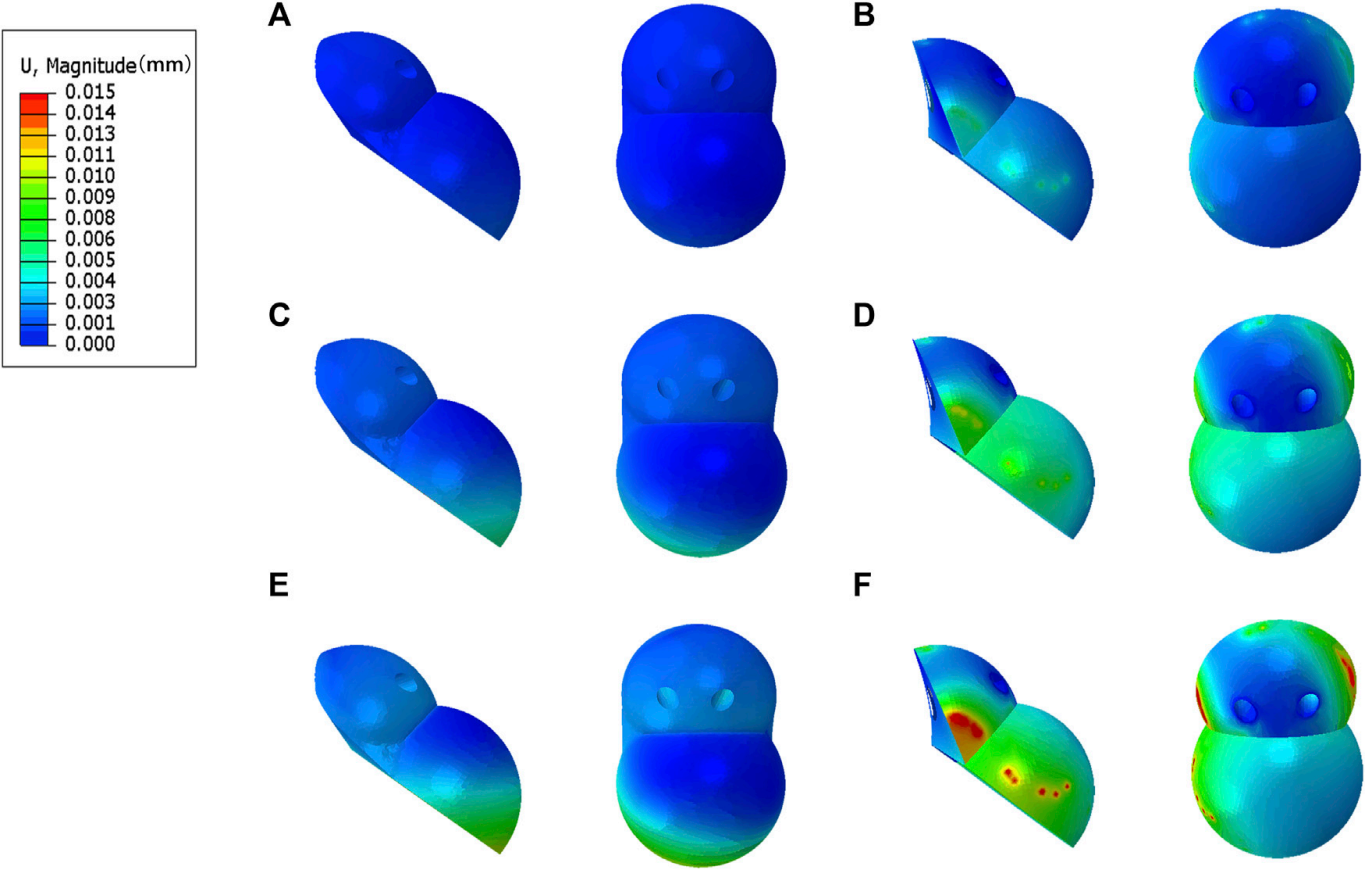
Relative micromotion distribution of the IAP and MAP relative to the host bone. **(A,B)** 210 N; **(C,D)** 2100 N; **(E,F)** 4200 N.

The peak micromotion of the IAP relative to the host bone was 1.5 μm (210 N), 8.9 μm (2100 N), and 11.2 μm (4200 N) (Figures 6A, C, E). The peak micromotion of the MAP relative to the host bone was 9.7 μm (200 N), 13.7 μm (2100 N), and 45.2 μm (4200 N) (Figures 6B, D, F).

### 3.5 Clinical follow-up results of patients

**FIGURE 7:**
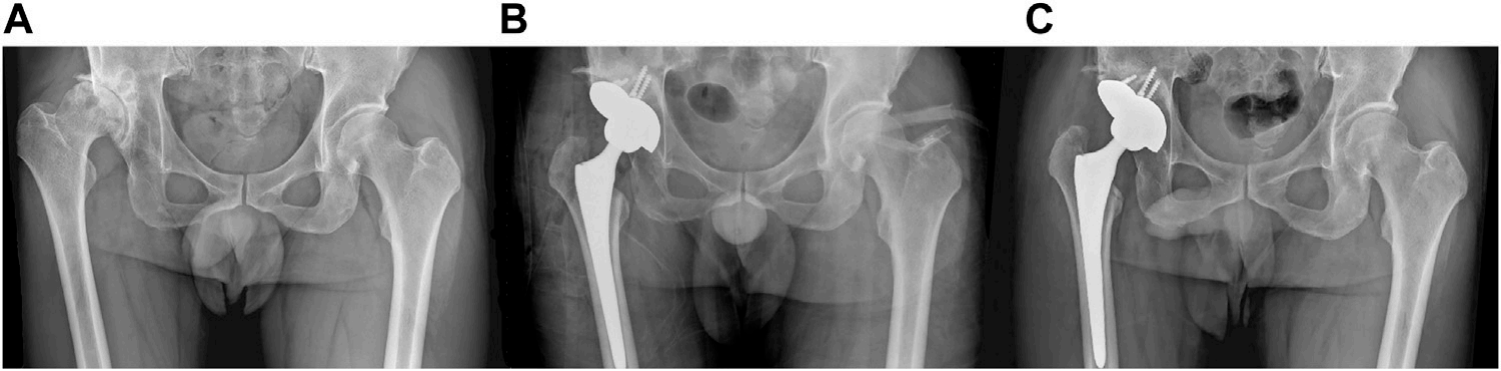
A 65-year-old male patient with Crowe III DDH who underwent 3D-printed IAP acetabular reconstruction. **(A)** Preoperative X-ray image; **(B)** immediate postoperative X-ray image; **(C)** postoperative last follow-up X-ray image.

The follow-up time for patients was 11.8 ± 3.4 months. The preoperative Harris hip score was 46.5 ± 4.8, increasing to 80.1 ± 6.6 at 3 months postoperative and 85.4 ± 5.5 at the final follow-up. No surgical site swelling, infection, or postoperative complications were observed during the last follow-up. In addition, all patients’ anteroposterior projection X-ray of the hip showed no adverse conditions such as radiolucent lines, loosening, and osteolysis around the 3D-printed IAP and bone surface (Figures 7A–C).

## 4 Discussion

The lack of adequate acetabular bone coverage on the superolateral part of the acetabular cup during the THA of Crowe III DDH will affect the initial stability of the acetabular cup (Crowe et al., 1979). The selection of an appropriate acetabular reconstruction method is the key to ensuring the biomechanical stability of the acetabular cup. The 3D printing technology provides a new choice for acetabular reconstruction in Crowe III DDH patients (Zhang et al., 2020). The IAP and MAP designed by the 3D printing technology can not only restore the rotation center of the hip joint, leg length discrepancy, and the muscle tension around the hip joint but also provide a stable and practical support for the acetabular cup to keep it in an ideal position. However, there is a lack of biomechanical research comparing the stability of the interface between the IAP and MAP acetabular components and the host bone.

To help clinicians better understand hip biomechanics and prevent complications, the FE analysis has been widely used in orthopedic implant design and preoperative planning. Compared to other experiments, it can not only simulate the biomechanical performance of implants as prostheses with good fidelity but also demonstrate higher efficiency and conserve resources (Fallahnezhad et al., 2023). This study used the FE analysis to evaluate the biomechanical advantages and disadvantages of the IAP and MAP by assessing the contact stress and relative micromotion between the acetabular prosthesis and the host bone under different loads.

**FIGURE 8:**
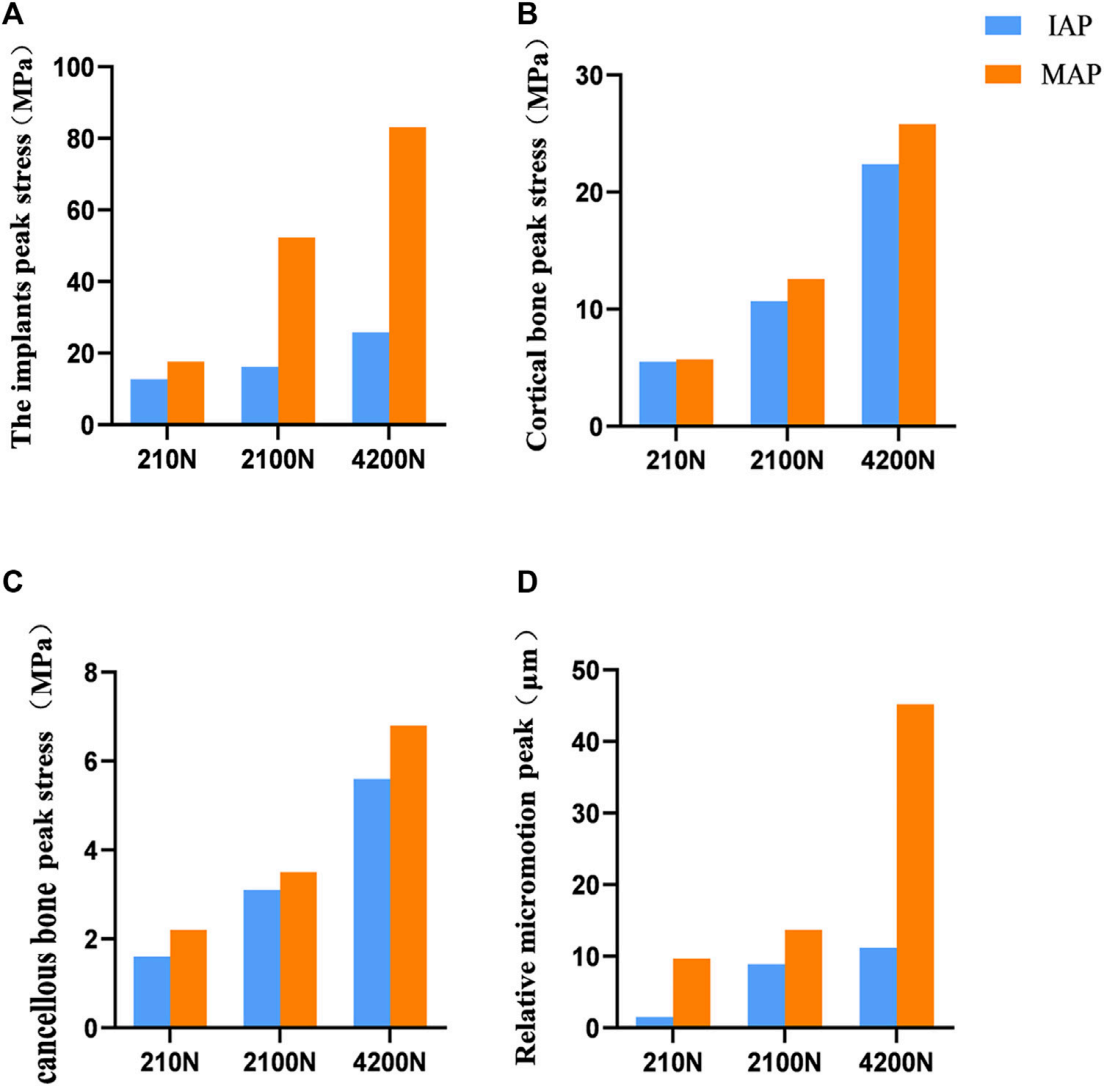
Comparison of contact stress and relative micromotion between the IAP and MAP. **(A)** Peak stress of the implant. **(B)** Peak stress of the cortical bone. **(C)** Peak stress of the cancellous bone. **(D)** Peak micromotion of the acetabular prosthesis relative to the host bone.

The acetabular cup and metal augment used in the present study were porous tantalum materials. The yield strength of porous tantalum has been reported to be 35–51 Mpa (Wang et al., 2023). Under the maximum load, the peak stresses of the IAP and MAP as porous tantalum implants were 25.8 Mpa (contacting the edge of the cortical bone) and 54.2 MPa (at the nail hole of the metal augment), and the peak stress of the MAP was more significant than the yield strength of porous tantalum. Therefore, the MAP is prone to failure and fracture when it reaches the exercise load immediately after operation. At the same time, under the same load, compared with MAP acetabular reconstruction, IAP, as an implant, had more uniform force and lower peak stress, which reduced the risk of prosthesis fracture (Figure 8A).

The mean yield strengths of cancellous and cortical bones near the acetabulum was 3.3 and 93.4 Mpa, respectively (Fu et al., 2018). Under a double-legged standing load, the peak interface stress between the cortical bone and the MAP was 5.7 MPa, and for the cancellous bone, it was 2.2 MPa. Similarly, the interface peak stress between the cortical bone and the IAP was 5.5 MPa, and for the cancellous bone, it was 1.6 MPa. Notably, the peak stresses at the interface of cortical and cancellous bones in contact with both the MAP and IAP were found to be below their respective yield strengths. According to the aforementioned data, the bone strength around the acetabulum is strong enough to support the patient to stand after acetabular reconstruction. Furthermore, the research has shown that a relative micromotion of less than 40 μm between the acetabular prosthesis and the host bone promotes bone ingrowth, which is beneficial for the long-term stability of the acetabular prosthesis (Kaku et al., 2015). Under a double-legged standing load, the relative micromotion of the MAP and IAP was 9.7 and 1.5 μm, respectively, which facilitated bone ingrowth, indicating that the two acetabular prostheses were stable under the standing load.

Under the exercise load, the peak interface stress of the MAP in walking and jogging contact with the cancellous bone was 3.5 and 6.8 Mpa, respectively, which were higher than the yield strength of the cancellous bone. However, the peak stress (5.6 Mpa) of the IAP exposed to the cancellous bone only exceeded the yield strength of the cancellous bone under the jogging load (Figures 8B, C). Therefore, compared with IAP acetabular reconstruction, the MAP cancellous bone has a higher risk of failure, and prosthesis fixation is unreliable and prone to loosening. Under jogging load, the micromotion of the MAP relative to the host bone was more than 40 μm, which was not conducive to bone growth and affected the long-term stability of the acetabular prosthesis. In contrast, the relative micromotion of the IAP to the host bone under three different loads was much smaller than the critical condition of bone growth, and the condition of bone growth was better, which was beneficial to the long-term stability of the acetabular prosthesis (Figure 8D). Therefore, according to the results of FE analysis, the IAP is safer than the MAP in acetabular reconstruction.

In summary, during the early postoperative exercise, with the high stress in MAP nail holes and MAP metal screws, the prosthesis has a higher risk of fracture and loosening. In contrast, the IAP not only effectively disperses the stress on the screws, reducing high-stress areas between the prosthesis and the host bone, thus lowering the risk of prosthesis fracture and loosening, but also provides more favorable conditions for bone ingrowth, promoting the long-term stability of the prosthesis. Regarding the reasons behind such biomechanical differences, we believe that in Crowe III DDH acetabular reconstructions, the integrated design of the IAP can effectively achieve uniform stress transfer in the acetabular prosthesis. Additionally, the fixation of multiple metal screws also provides enhanced stability for the IAP. On the contrary, the multi-component design of the MAP makes the acetabular prosthesis stress distribution uneven, and the contact between the components will cause the prosthesis to loosen due to the change in biomechanical load. Therefore, from the biomechanical perspective, the IAP reconstruction of the acetabulum can provide more excellent biomechanical properties while maintaining reliable structural strength.

In clinical applications, a high loosening and fracture rate of the acetabular prosthesis after follow-up for acetabular defect reconstruction using the MAP was also reported in the literature Borland et al. (2012); Cassar-Gheiti et al. (2021). In this study, five Crowe III DDH patients undergoing IAP acetabular reconstruction were clinically followed up. The hip function of all patients was significantly improved, and the quality of life of patients was greatly improved. The imaging results showed that the acetabular prosthesis is firmly fixed with no signs of loosening or fracture observed, indicating the satisfactory stability of the prosthesis. This clinical result has further confirmed the validity of the biomechanical results of this study.

The limitations of this study are as follows: 1) The influence of muscles and soft tissues around the hip joint was not considered in this study: only hip contact forces were used for testing, which might not accurately reflect hip joint motion under physiological loading patterns. 2) The results of this study were generated based on computer simulations and were not validated using cadaveric bone for biomechanical studies. 3) No clinical case comparison was performed primarily because the FE analysis results of the MAP model indicated higher clinical application risks. Therefore, clinical validation was carried out exclusively on IAP cases. 4) This study merely referenced prior research for mesh configuration and did not perform mesh sensitivity analysis. Despite these limitations, our findings may help orthopedic surgeons to select a more appropriate acetabular reconstruction method in clinical practice.

## 5 Conclusion

In this study, the biomechanics of the IAP- and MAP-reconstructed Crowe III DDH acetabulum designed by 3D printing technology were evaluated by the FE analysis. The results show that the risk of loosening and fracture of the prosthesis is lower and the long-term stability is better with the IAP than with the MAP reconstructed acetabulum, suggesting that the IAP may have more excellent biomechanical properties than the MAP in Crowe III DDH acetabular reconstruction. Clinical follow-up of five patients with Crowe III DDH acetabular reconstruction by IAP showed good clinical efficacy, which has further verified the effectiveness of the IAP reconstruction of the acetabulum. These results can provide a biomechanical reference for the selection of clinical treatment.

## Data Availability

The original contributions presented in the study are included in the article/Supplementary Material. Further inquiries can be directed to the corresponding authors.
